# The Influence of Environmental Factors on the Degradation of PLA/Diatomaceous Earth Composites

**DOI:** 10.3390/polym16111450

**Published:** 2024-05-21

**Authors:** Marta Dobrosielska, Renata Dobrucka, Dariusz Brząkalski, Martyna Pajewska-Szmyt, Krzysztof J. Kurzydłowski, Robert E. Przekop

**Affiliations:** 1 Faculty of Materials Science and Engineering, Warsaw University of Technology, ul. Wołoska 141, 02-507 Warsaw, Poland; marta.dobrosielska@pw.edu.pl; 2 Department of Non-Food Products Quality and Packaging Development, Institute of Quality Science, Poznań University of Economics and Business, al. Niepodległości 10, 61-875 Poznań, Poland; 3 Centre for Advanced Technologies, Adam Mickiewicz University in Poznań, ul. Uniwersytetu Poznańskigo 10, 61-614 Poznan, Poland; dbrzakalski@gmail.com (D.B.); mpszmyt@amu.edu.pl (M.P.-S.); 4 Faculty of Mechanical Engineering, Bialystok University of Technology, ul. Wiejska 45c, 15-351 Bialystok, Poland; krzysztof.kurzydlowski@pb.edu.pl

**Keywords:** polylactide, diatomaceous earth, diatomite, silanes, chemical modification, degradation, mechanical properties

## Abstract

In the present study, tests were carried out on composite samples on a polylactide matrix containing 25% by weight of mineral filler in the form of diatomaceous earth, base, and silanized with GPTMOS (3-glycidoxypropyltrimethoxysilane), OTES (n-octyltriethoxysilane), and MTMOS (methyltrimethoxysilane) silanes. The addition of two types of waxes, synthetic polyamide wax and natural beeswax, were used as a factor to increase the rheological properties of the composites. The obtained samples were characterized in terms of the effect of filler silanization on the degradation rate of the composites. The tests were conducted under different conditioning conditions, i.e., after exposure to strong UV radiation for 250 and 500 h, and under natural sunlight for 21 days. The conditioning carried out under natural conditions showed that the modified samples exhibit up to twice the degradation rate of pure polylactide. The addition of synthetic wax to the composites increases the tendency to agglomerate diatomaceous earth, while natural wax has a positive effect on filler dispersion. For composites modified with GPTMOS and OTES silanes, it was noted that the addition of natural wax inhibited the degree of surface degradation, compared to the addition of synthetic wax, while the addition of MTMOS silane caused the opposite effect and samples with natural wax degraded more strongly. It was shown that, despite the high degree of surface degradation, the process does not occur significantly deep into the composite and stops at a certain depth.

## 1. Introduction

The weather resistance of composite materials is an important feature from the point of view of the consumer and various industries. The composite should be characterized, depending on the needs of the target group, by an appropriate mechanical strength, including impact resistance or flexibility, a hydrophobic or hydrophilic profile of the surface, or color stability under the influence of environmental factors or the passage of time. Commonly known and used polymers or composites for structural applications are usually petroleum-based, which, due to their production process, makes them harmful to the environment. A good alternative is polymers obtained from monomers synthesized from raw materials obtained from renewable sources, such as polyamide 11 or polylactide. In addition to the environmental aspect, they are also characterized by physicochemical or mechanical properties acceptable for selected applications, especially when combined with suitable fillers, such as diatomaceous earth [1,2], wood flour [3] or various types of fibers (flax and jute [4], bamboo [5], and beech fibers [6]). Diatomaceous earth alone, as shown in previous works [7,8,9], can reinforce a composite based on polylactide [10] or polyamide 11, especially if it contains particles of the smallest size [8]. On the other hand, the elimination of these particles results in a composite that does not have adequate mechanical strength compared to the starting polymer [9].

According to the standard adopted by the EU, biodegradable plastic should be converted to carbon dioxide, water, and minerals by more than 90% in a maximum of six months. The biodegradation of polylactide itself takes place under biological conditions with the participation of fungal or algal bacteria, which use the polymer as a food source [11]. Biodegradable polymers should remain unchanged for the lifetime of the polymer, and then biodegrade. The degradation process of PLA itself begins at the surface of the polymer, and then occurs throughout the plastic. PLA can undergo photodegradation, thermal degradation, oxidative degradation, bacterial degradation, enzymatic degradation, and hydrolytic degradation, but, usually, scientific work focuses on the last two modes [12]. The process of hydrolytic degradation can occur under the influence of not only water, but also aqueous acetonitrile solutions, aqueous alcohol solutions, or alcohols (alcoholysis) [13,14,15]. During hydrolysis, the ester groups of the polymer chain are broken down, the polymer being converted into soluble oligomers and monomers, and the ester bonds are fragmented, leading to a decrease in the average molecular weight of the polymer [16].

In addition to atmospheric precipitation, which contributes to hydrolytic degradation, polylactide in structural applications is exposed to ultraviolet radiation, which is destructive to any organic material, which, in turn, starts the process of photolytic degradation, which, in turn, contributes to the cracking of the material or color change, which negatively affects the visual aspects [17,18]. Diatomaceous earth has been proven to increase the degradation rate of a polylactide-based composite [19]. Therefore, in the present work, we have attempted to develop a composite based on polylactide and silanized diatomaceous earth, resistant to both hydrolytic and photolytic degradation. From the point of view of ecology, the deliberate pursuit of a short life of the composite or polymer and rapid degradation under natural conditions is not beneficial due to the energy consumption in the production of the product or, despite the use of biocomposites, environmental pollution by the products of their decomposition, especially carbon dioxide. For this reason, attempts to produce a composite for structural applications appear to address the need to reduce the carbon footprint and pursue the concept of a circular economy, and could potentially contribute to reducing global warming by reducing CO_2_ emissions into the atmosphere. To this end, polylactide-based composites filled with silanized diatomaceous earth were fabricated, and then aged under simulated (UV-aging chamber) and natural conditions for an appropriate period of time. After conditioning, among other things, the degree and depth of degradation of the composites were examined, and visual tests were carried out, including the study of color change under UV light. Various methods of degradation of composites, both laboratory and natural, are described in the literature. Polylactide matrix composites modified with mineral fillers have been conditioned under natural conditions in acidic soil [19], abiotic hydrolysis, biotic degradation, and composting conditions in a laboratory environment [20] or under conventional weathering conditions [21]. The degradation conditions we proposed combine both simulated and natural conditions, which allowed us to study the influence of photolytic and hydrolytic factors on the degradation of polylactide. As for the application in research on diatomaceous earth polylactic acid complexes, they are in early development. The aim of our research was the impact of the weather conditions on the material. The aim of our research is to develop polylactide-based composites for use in products that do not come into contact with food. Most of the research focuses on the use of PLA in packaging. However, we see a problem related to plastic elements that are used as elements of, for example, textiles and clothes—buttons, clothes zippers, and other small elements used in home interiors, e.g., switches, and kitchen elements—which can be unintentionally or consciously transferred to the environment. The developed material is, therefore, supposed to have good design features during its use and be characterized by rapid degradation when released into the environment. However, we believe that the development of a material that, if accidentally released into the environment, can degrade more quickly and harmlessly may be important. Moreover, we wanted to test whether polylactide could degrade faster under environmental conditions without the participation of bacteria or composting conditions.

## 2. Materials and Methods

### 2.1. Materials

Polylactide (PLA)-type Ingeo 4043D was purchased from NatureWorks (Minnetonka, Minneapolis, MN, USA). Diatomaceous earth (Perma-Guard, Bountiful, UT, USA) was derived from diatomite deposits. Synthetic wax WTH-B microbeads (behenamide, CH_3_(CH_2_)_20_CONH_2_) were bought from WTH GmbH (Hamburg, Germany). Beeswax was derived from Spółdzielnia Pszczelarska APIS (Lublin, Germany). Silanes GPTMOS (3-glicydoksypropylotrimetoksysilan) OTES (n-oktylotrietoksysilan) and MTMOS (metylotrimetoksysilan) were purchased from Sigma-Aldrich (Saint Louis, MO, USA).

### 2.2. Preparation of Modified Diamaceous Earth

First, 1 kg of diatomaceous earth was placed in a 20 L glass reactor equipped with a mechanical stirrer. Then, 4 L of iPrOH, 10 mL of demineralized water and 1% *w*/*w* of the appropriate silane (GPTMOS, OTES, MTMOS) were added to the reactor and left for two hours with constant stirring (300 rpm). After 2 h, 10 mL of concentrated hydrochloric acid (HCl) was added in portions. After 24 h of continuous stirring, the mixture was transferred to a separate container and left to settle. After pouring off the liquid from the sediment, the diatomaceous earth was washed with demineralized water and placed in an oven at 60 °C for 24 h. The silanes used for modification were GPTMOS (3-glycidoxypropyltrimethoxysilane), OTES (n-octyltriethoxysilane), and MTMOS (methyltrimethoxysilane).

### 2.3. Preparation of Granulates

PLA-based composites were prepared using a ZAMAK MERCATOR WG 150/280 laboratory rolling mill (Skawina, Poland). For this purpose, 250 g of PLA 4043D Ingeo at 210 °C was mixed together with 250 g of modified or base diatomaceous earth and 2 or 4% by weight of natural (beeswax) or synthetic (WTH-B) wax, respectively. The process was carried out until homogeneous mixtures were obtained. The resulting systems were then ground using a WANNER C17.26 sv mill and dried for 24 h at 60 °C. Systems containing unmodified diatomaceous earth were also prepared.

### 2.4. Preparation of Samples for Measurements

The samples were diluted 1:1 with PLA directly in the injection molding machine. Table 1 shows the process parameters. A holding pressure of linear increment over 9 s time was applied. The mold temperature was maintained at 80 °C. Standardized molds for mechanical testing in accordance with PN-EN ISO 20753:2019-01 [22] were obtained. The final concentrations of the systems are shown in Table 2.

### 2.5. Characterization Methods

For tensile strength test, the materials obtained were injection-molded as type 1B dumbbell specimens in accordance with EN ISO 527:2012 [23] and EN ISO 178:2006 [24]. Tests of the obtained specimens were performed on a universal testing machine INSTRON 5969 (Norwood, MA, USA) with a maximum load force of 50 kN. The traverse speed for tensile strength measurements was set at 2 mm/min. Seven samples of each type were tested. Color change (∆E) was measured using an NR60CP colorimeter in 8°/d geometry, SCI. The difference between two colors in CIELab space was calculated using the formula:

$$\Delta E=\sqrt{{\left(\Delta L\right)}^{2}+{\left(\Delta a\right)}^{2}+{\left(\Delta b\right)}^{2}}$$

where ∆L = L_1_ − L_2_; ∆a = a_1_ − a_2_; and ∆b = b_1_ − b_2_.

Gel permeation chromatography was performed using an Agilent Technologies 1260 liquid chromatograph equipped with a refractive index detector. The mobile phase was tetrahydrofuran (THF), while the stationary phase was Phenogel 10 µ Linear(2) 300 × 7.8 mm (100–10,000,000 g/mol). A flow rate of 1 mL/min was used, the column was maintained at 35 °C, and a polystyrene standard (MW range—1310–3,640,000 g/mol) was used to prepare a standard curve. The sample for GPC testing was taken by scraping a layer of investigating material with a cutter and weighing 10–13 mg in glass vial. Next, the samples were suspended in tetrahydrofuran (THF), ultrasonicated at 35 °C until dissolved, and then filtered through a 0.2 μm-pore-size filter. The degradation degree was calculated from the changes in M_w_ parameter. Images of composite surfaces and fractures were taken using a KEYENCE VHX-7000 digital microscope (Keyence International NV/SA, Osaka, Japan) with a VH-Z100R wide-angle zoom lens with 100× and 1000× magnification. The images were taken using the depth composition and 3D image creation functions. Total coaxial illumination was used.

Composite conditioning was carried out in an Atlas UV Test aging chamber with 313 nm UV-B fluorescent lamps. The test was performed in accordance with ISO 4892-3 [25], test cycle 2 according to ASTM G154 [26], and PN-EN ISO 16474-3 [27]:

− UV irradiation—intensity of radiation 0.71 W/m^2^, 60 °C—4 h (light segment);
− condensation 50 °C—4 h (dark segment).

Aging test was conducted for 250 and 500 h.

**Figure 1:**
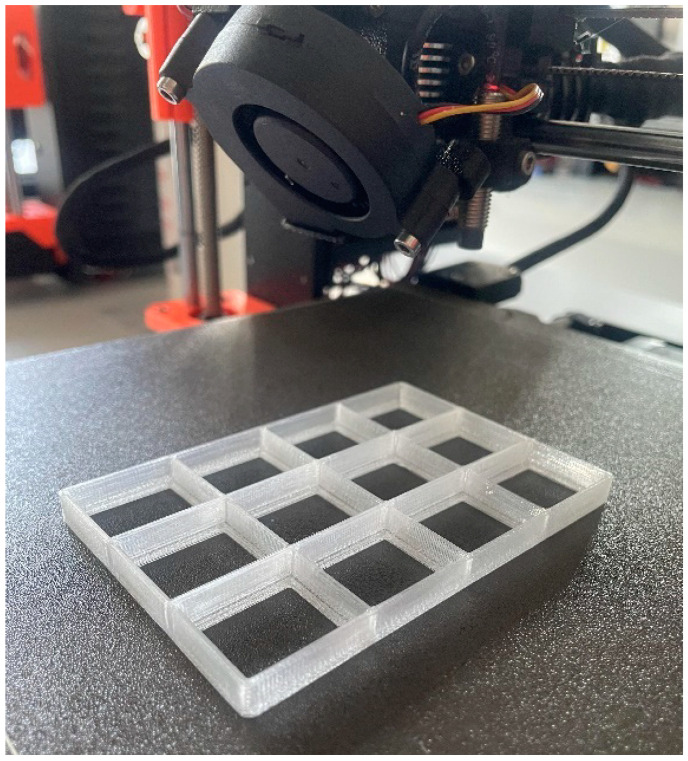
UV-aging sample holder.

Samples for aging tests in the UV chamber were placed in custom-made holders, measuring 66 × 98 mm, printed from PET using FDM 3D printing. The holder has 12 spots measuring 20.0 × 23.0 × 6.4 mm. The wall thickness between the sample spots is 1.8 mm. A photo of the holder is shown in Figure 1. The composite samples were prepared in the form of cubes with dimensions corresponding to those of the spots available in the holder.

**Figure 2:**
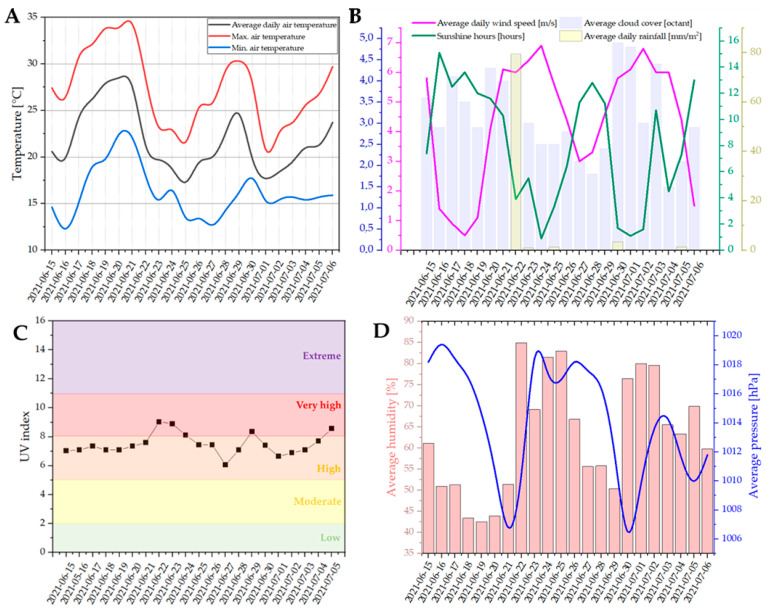
Average daily air temperature, and maximum air temperature and minimum air temperature (**A**); average daily wind speed, average cloud cover, sunshine hours, and average daily rainfall (**B**); UV index (**C**); and average humidity and average pressure (**D**) between 15 June 2021 and 6 July 2021.

Conditioning under natural sunlight was carried out from 15 June 2021 to 6 July 2021, for a total of 21 days (~500 h). The composite samples were placed in an unshaded location. Measurement data of weather conditions occurring during the conditioning of the samples were obtained from the meteorological station Poznań-Ławica with geographical co-ordinates 52°25′ 16°50′. Meteorological data are presented as follows: average daily air temperature, and maximum and minimum daily air temperature, are summarized in Figure 2A; average daily cloud cover, average daily wind speed, average daily rainfall, and number of hours of sunshine are summarized in Figure 2B; average UV index is summarized in Figure 2C; and average monthly humidity and average atmospheric pressure are presented in Figure 2D.

## 3. Results

### 3.1. Mechanical Tests

**Figure 3:**
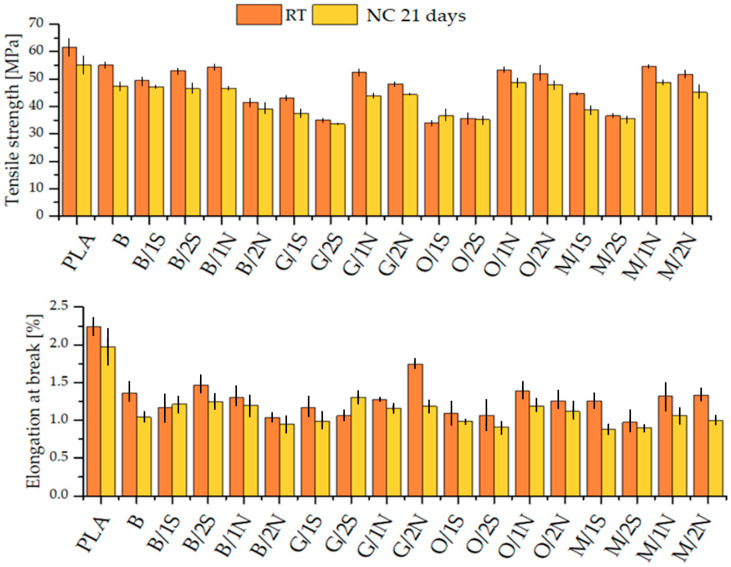
Tensile strength and elongation at break of the composites in as-received state and after aging under natural conditions.

Tensile strength tests were conducted on samples aged under natural conditions. After 21 days, the tests showed a slight decrease in both tensile strength and elongation at tension for all analyzed samples, including pure polylactide (Figure 3). The use of synthetic wax in composites with DE-silanized OTES and GPTES resulted in a decrease in the strength of the composites, indicating the limited compatibility of the filler prepared in this way with the wax. The effect of the compatibility of synthetic and natural waxes with diatomaceous earth subjected to surface modification with various silane coupling agents was discussed in detail in an earlier paper [28]. This work is a continuation of the previous paper. The decrease in mechanical strength is related to the degradation of neat PLA present in each analyzed system and comparable for most systems; however, the lowest is for composites with synthetic wax in combination with the silanized filler, indicating the greatest stability of the systems, probably due to the combined effects of the presence of a well-dispersed filler as a UV blocker and hydrophobic wax stopping the composite from moisture diffusion. On the other hand, the mentioned systems provided the poorest mechanical properties as received, showing the limited compatibility of synthetic wax with the remaining components, especially the PLA matrix, as proven by the previous study [29]. Moreover, the polymer degradation occurs mostly in the near-surface region, which is further explained in the following sections (see Section 3.2 and Section 3.4). The elongation at break was similar for most of the composites and was reduced in comparison to neat PLA due to the presence of the particulate filler.

### 3.2. Degradation of PLA Macromolecular Structure

**Figure 4:**
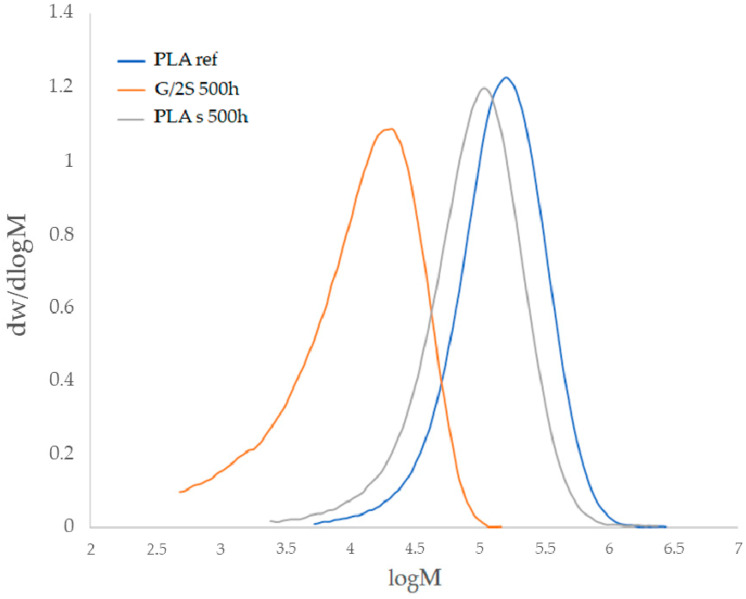
MWD curve of polylactide conditioned at room temperature and at 500 h in an aging chamber and G/2S-modified composite conditioned at 500 h in an aging chamber.

**Figure 5:**
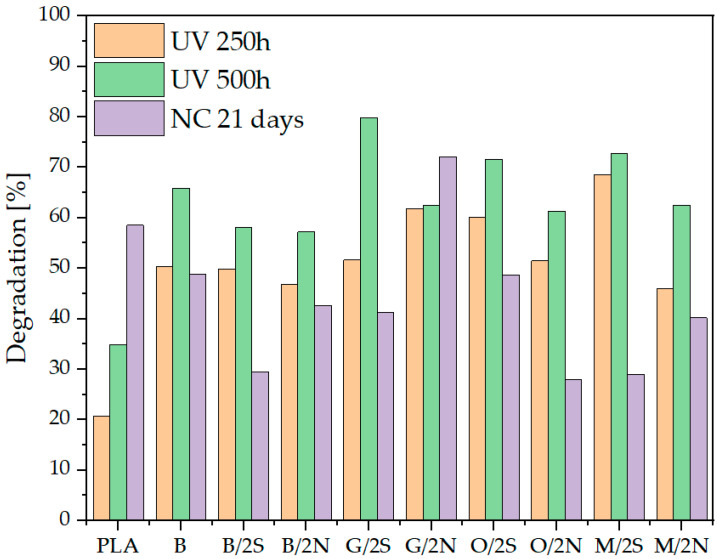
Determined chain degradation for the polymer samples tested.

Degradation tests were carried out for both the composite samples placed in the UV-aging chamber and the samples exposed to natural weathering for 21 days. The analysis was performed on the outer layer of the composite, as both a mechanical analysis (see Section 3.1) and a microscopy analysis (see Section 3.4) provided the evidence on the polymer degradation occurring mostly on the near-surface layer. The polymer degradation was studied on the basis of the number average (Mn) and weight average (Mw) molecular weight (Table 3). Samples for GPC were collected from the surface of the sample oriented toward sunlight. The addition of the filler, as well as the use of processing additives, increased the degradation of the polymer, as can be observed from the MWD mass distribution curves (Figure 4), where the individual curves shift to the left relative to the reference sample, as well as in the designated degradation degree (Figure 5). For the PLA sample without modifiers, the degradation was 20.7%, 35.9%, and 58.5% for samples subjected to accelerated aging after 250 h and 500 h and during natural exposure, respectively. Modifications of the polymer increased the degradation of the samples, however, without any correlation between the exposure conditions. The highest percentage of chain degradation occurred for sample G/2S, i.e., 79.8%, after UV-500h exposure.

Neat polylactide degraded progressively over time after aging in a UV chamber (20.7% and 34.9% after 250 h and 500 h, respectively), and degraded 58.5% after conditioning under natural conditions. The modified composites for each test time degraded more under controlled conditions than the reference PLA, both with the addition of neat diatomaceous earth and diatomaceous earth (base or silanized) in combination with waxes. Under natural conditions, the differences between the degradation of neat PLA and the composites, as well as between individual composites, were characterized by too much statistical scatter to show a significant effect of fillers and waxes on the stability of the materials under natural conditions. The factors influencing the observed differences are threefold: First, the samples in the UV chamber are subjected to aging under standardized radiation and humidity conditions. Secondly, in the UV chamber, the samples are subjected to moisture during the aging cycle, during the dark condensation segment. The contribution of moisture significantly accelerates the plastic degradation process [30]. Finally, under the natural aging conditions, the UV irradiation is of a wide range, while, under the simulated conditions, it is provided by UV-B 313 nm lamps. Under natural conditions, the UV index was oscillating around 8 for most of the experiment, as taken from the meteorological data provided, which is considered to be an equivalent of 0.2 W/m^2^ of biologically effective UV irradiance; however, it depends on the calculation model and, therefore, cannot be simply compared to the total UV irradiance [31,32]. The last effect may also explain the significantly higher degradation of neat PLA under natural conditions when compared to the simulated ones. The above results suggest that the modification decreased the stability of the composites in an environment with a high proportion of moisture acting as a hydrolytic agent. This is related to the increased porosity of the material, introduced by the filler, which leads to the accelerated diffusion of water in the polymer and increases the contact area of water with the polymer phase. The addition of waxes, especially the synthetic one, slightly caused a decrease in the degradation of the polymer under natural conditions, which is related to the hydrophobicizing effect and the improved dispersion of the filler, translating into its effectiveness as a physical UV blocker. Under simulated conditions, on the other hand, it was noted for systems containing 2% wax that, each time, the synthetic wax caused a higher level of PLA degradation than the natural wax in the corresponding system. This was due to the high proportion of moisture and radiation intensity, leading to the degradation and elimination of wax from the composite structure.

**Figure 6:**
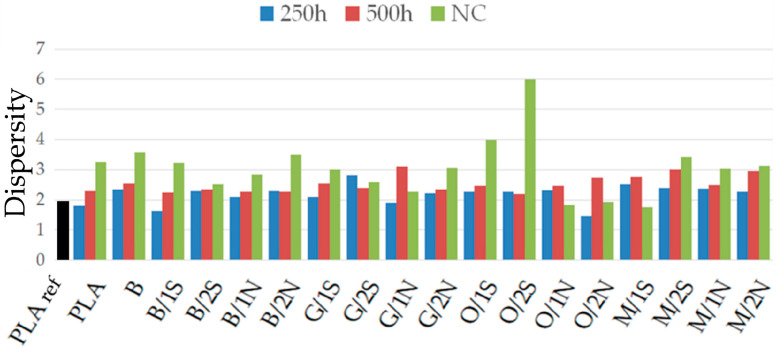
Determined dispersity index (Ð) for the tested samples, after different aging periods.

Based on the Mw/Mn ratio, the dispersity index (Ð, Figure 6), a measure of polymer chain length inhomogeneity, was determined. For the reference sample, it is about 1.95, while there is an increase in the index for samples subjected to UV and natural exposures, due to polymer chain breakage at statistically random locations. A significant increase in PDI for samples aged under natural conditions is noticeable. This is related to the occurrence of a less efficient mechanism of polymer degradation under environmental conditions. Under simulated conditions, the mechanism of polymer chain degradation involves water, which, in the form of water vapor and moisture, is able to penetrate the pores of the material and permeate the filler phase and diffuse through the amorphous phase of PLA [33], especially at an elevated test temperature (60 °C), resulting in the degradation of the composite in its depth and, thus, averaging the degree of degradation over the thickness of the sample, at a time when, under relatively dry conditions (natural conditions), the material is mainly exposed to UV radiation, which is retained by the filler acting as a UV blocker.

**Figure 7:**
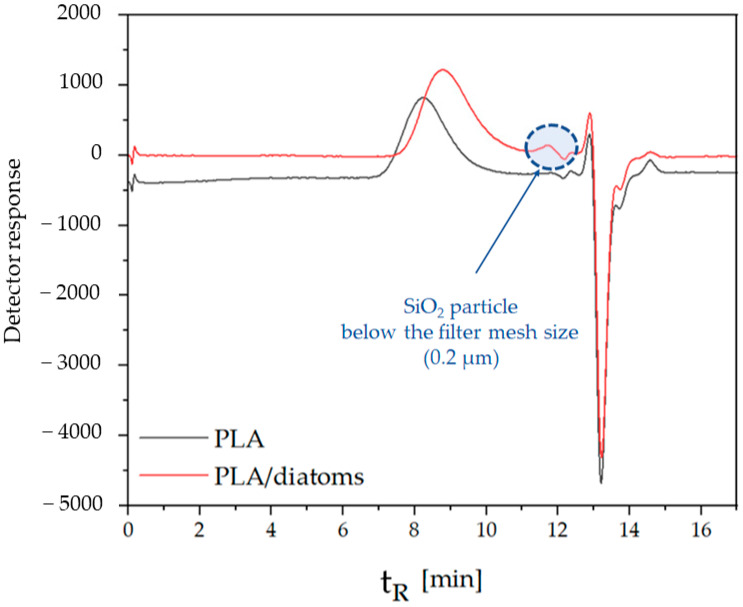
GPC-RI analysis for PLA sample without additive and with addition of diatomaceous earth.

**Figure 8:**
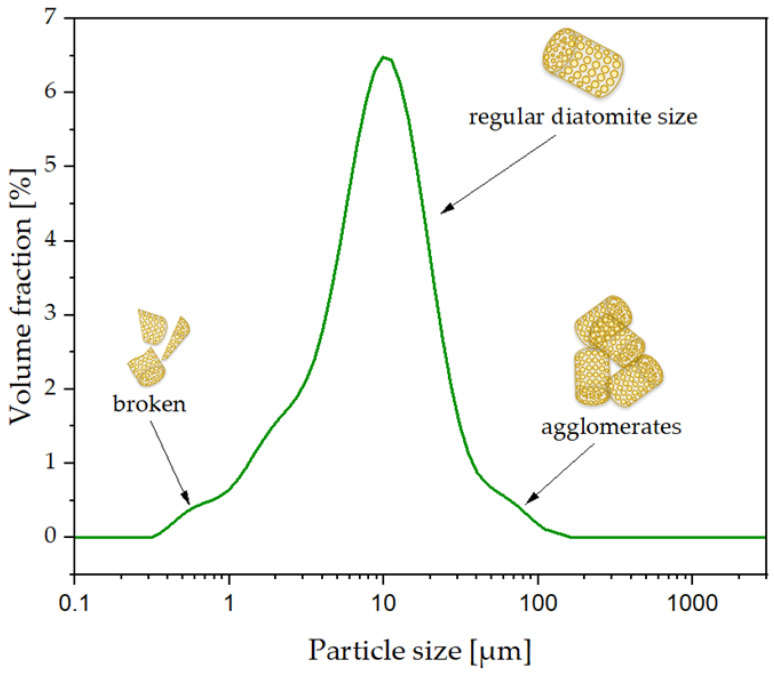
Particle size distribution of diatomaceous earth.

In the samples obtained from the aging of the composites, an additional peak (Figure 7) was observed at both 250 h and 500 h of degradation. It is suspected that its presence is related to the diatomaceous earth used for modification. Diatomaceous earth has particles in a wide size range, as illustrated in Figure 8. The DLS analysis of the filler used to prepare the PLA composites allowed the detection of particles with sizes whose lower size range was ~0.3 μm. The samples of aged composites were, after dissolution in THF, filtered through a membrane filter with a pore diameter of 0.2 μm. Considering the processing, which can lead to the crushing of filler particles, it can be suspected that the presence of the aforementioned peak is related to the content of particles with sizes below 0.2 μm. Magaletti and co-workers observed with GPC analyses of samples marine diatom Cylindrothece fussiformis low-molecular-weight peaks (low-molecular-weight peaks) of exopolysaccharides, and it can also be suspected that the emerging peak (tR 11.7 min) may be related to compounds from the polysaccharide group released from diatoms [31].

### 3.3. Color Testing after UV Irradiaton

**Figure 9:**
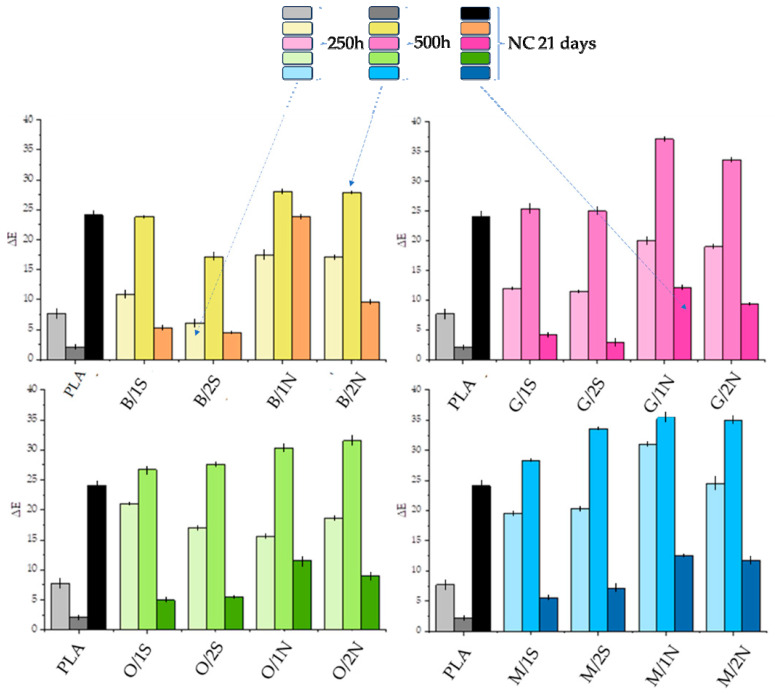
Differences in color of composites conditioned in an aging chamber for 250 and 500 h and under natural conditions.

**Figure 10:**
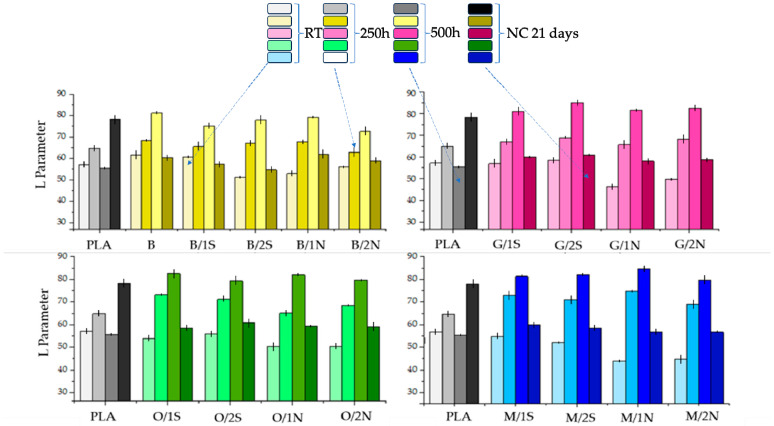
Luminance of composites conditioned in an aging chamber for 250 and 500 h and under natural conditions.

Figure 6 shows the differences in color (ΔE) of the different composites after exposure to both UV radiation and natural environmental conditions (NC). The color change was measured against baseline samples conditioned under room conditions. Significant and noticeable color changes were observed for all systems. Note that a color change according to the CIELAB color space scale, exceeding a value of 5, indicates a change that is significant and visible to the naked eye. The smallest color differences were observed for systems conditioned under natural sunlight, except for pure polylactide, for which ΔE was as high as ~25 (Figure 9). Compared to baseline PLA, systems filled with diatomaceous earth with the addition of synthetic wax showed improved color stability under natural conditions, while worse under simulated conditions. Natural wax caused a stronger color change with aging than synthetic wax, especially under natural conditions. Silanization of diatomaceous earth, especially OTES, led to an intensification of the color change effect, which may be explained by a reduction in wax concentration in the filler-polymer interphase and its easier migration to the plastic surface. An increase in the L parameter (CIELab method) indicates an increase in brightness of the material. In the case of the analyzed samples, the change in color each time consists in the discoloration of the sample under the influence of sunlight and a change to a whiter color. Considering the samples conditioned in the UV chamber for 250 h and 500 h, the color change for composites filled with diatomaceous earth is expected, i.e., the longer the time spent in sunlight, the greater the change in the L parameter (Figure 10). No significant differences were found between the systems, either due to the type of silane used or the type of wax and its concentration. The largest color change toward white was observed for the composites after 500 h in the UV chamber (L in the range of 80–90), while after 21 days under natural conditions, the swap is much smaller, as the L parameter is around 60.

**Figure 11:**
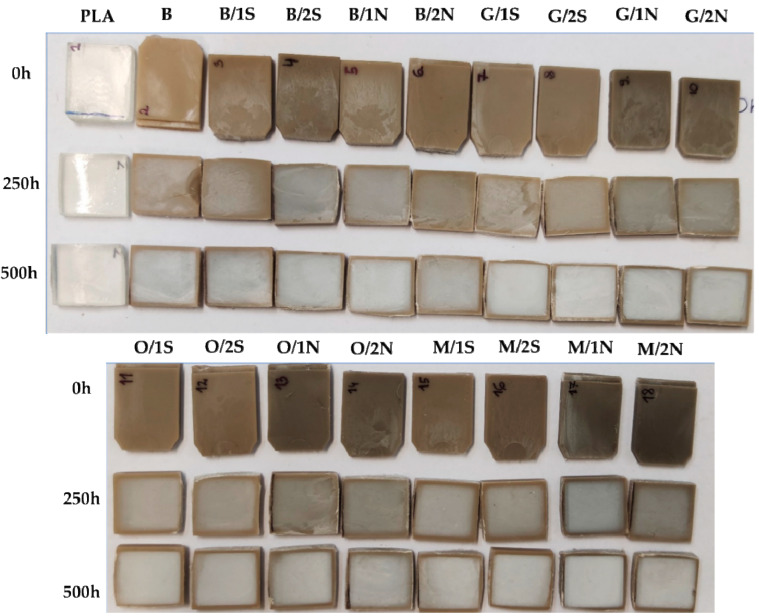
Composites before and after conditioning in a UV-aging chamber for 250 and 500 h.

Depending on the type of silane used or the type and concentration of the rheological agent (wax), the composites are characterized by differences in color (Figure 11), which was also found in colorimetric studies (Figure 9 and Figure 10). After conditioning the composites for 250 h in a UV chamber, their surface became visually bleached. Conditioning for 500 h in a UV chamber the effect deepened, a stronger bleaching of the surface was visible, however visual inspection did not confirm the presence of cracks or other defects. The pure polylactide, initially transparent, became dull and slightly yellowed under UV irradiation The energy transferred to the sample by means of heat and UV irradiation, as well as degradation-induced decrease in the average molecular weight may induce amorphous PLA phase crystallization and thus increase of crystalline phase content, especially near the sample surface, resulting in the sample opacity. The effects of PLA crystallinity during degradation have been discussed by others [34,35].

### 3.4. Microscopy Analysis of the Composites’ Structure after UV Irradiaton

**Figure 12:**
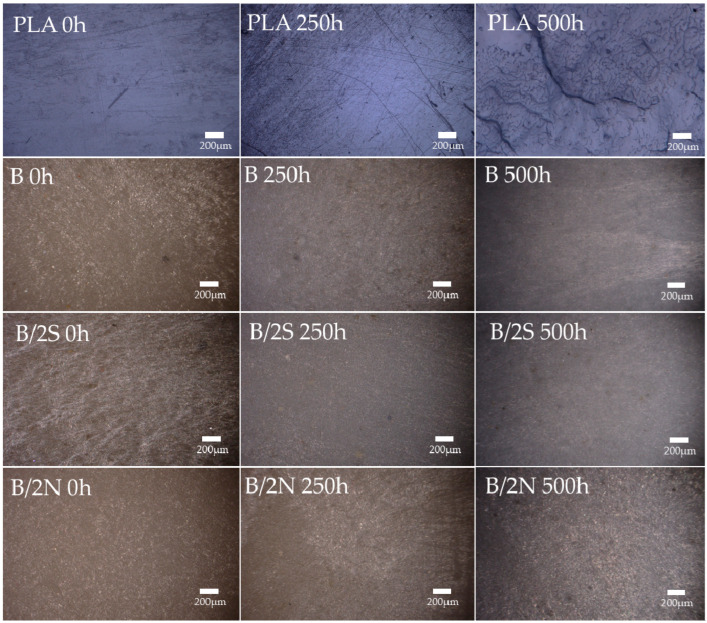
Surfaces of composites before and after placement in UV chamber (250 h and 500 h); systems without silanization.

**Figure 13:**
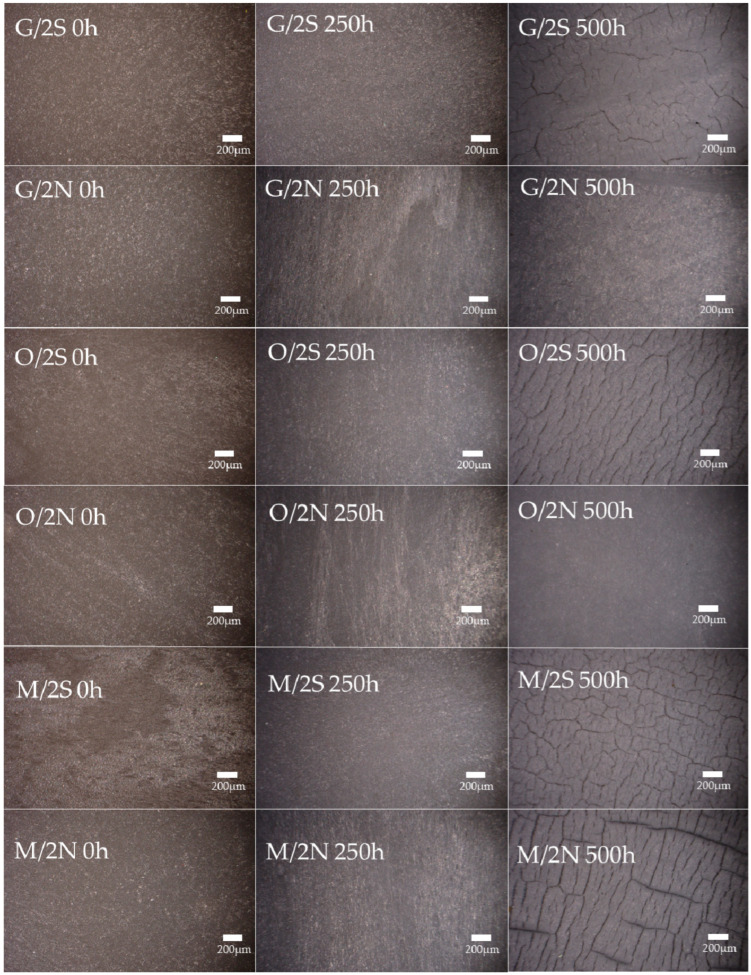
Surfaces of composites before and after placement in UV chamber for 250 and 500 h; silanized systems.

An analysis of the composites before the exposure to degradation agents confirmed the homogeneous structure of their surfaces (Figure 12 and Figure 13); no defects in the structure are noticeable. Placing the composites in the UV chamber for as short a time as 250 h caused both the transformation of the PLA matrix (most likely the crystallization mentioned earlier) and the migration of the wax on the surface of the samples, especially for all composites modified with natural wax, which is visible in the form of white, uneven discoloration on the surface of the composite. For samples containing synthetic wax, the effects of wax migration were negligible, with discoloration caused by changes in the PLA matrix. On the surface of pure PLA, after an identical time spent in the aging chamber, fine cracks are visible, which did not occur on the surface of the composites. Its further exposure to unfavorable conditions increased the cracks formed earlier and significant defects were observed in the structure. The synthetic wax-modified composites behaved similarly. The G/2S, O/2S, and M/2S systems are characterized by significant surface cracks, while, in the case of the silanized filler GPTMOS and OTES, analogous samples containing beeswax have no cracks and their surface is smooth with no visible defects. Regarding the GPC analysis of the material from the surface of the composites, the aforementioned systems with synthetic wax were, each time, characterized by a higher degradation rate than those with natural wax, which indicates the differences in the degradation rate of these composites and the lack of effect of the synthetic wax protecting the composite surface from degradation.

**Figure 14:**
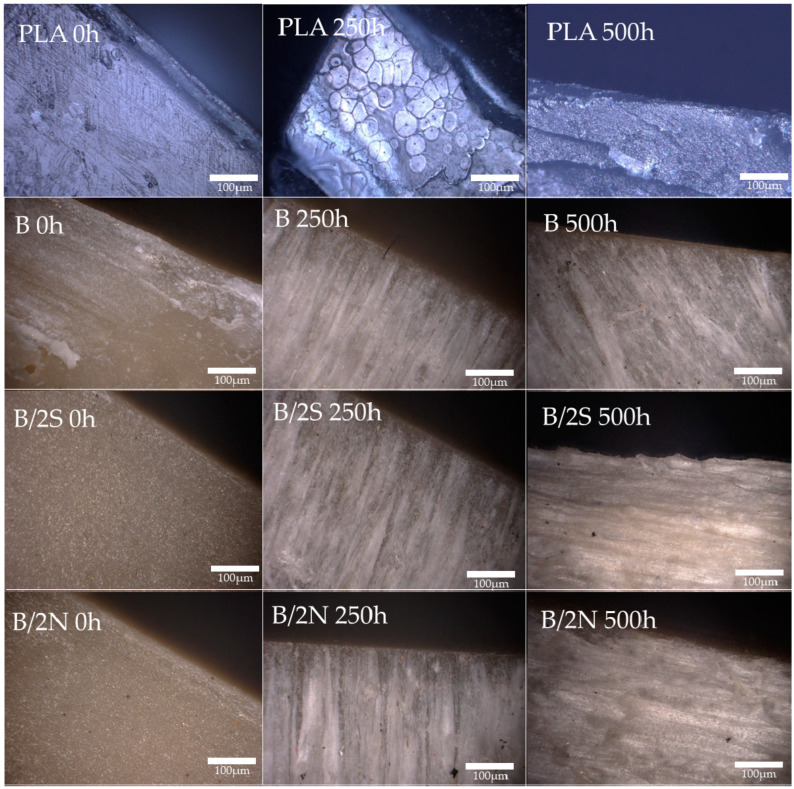
Edge structures of composite breakthroughs before and after placement in UV chamber (250 h and 500 h); systems without silanization.

**Figure 15:**
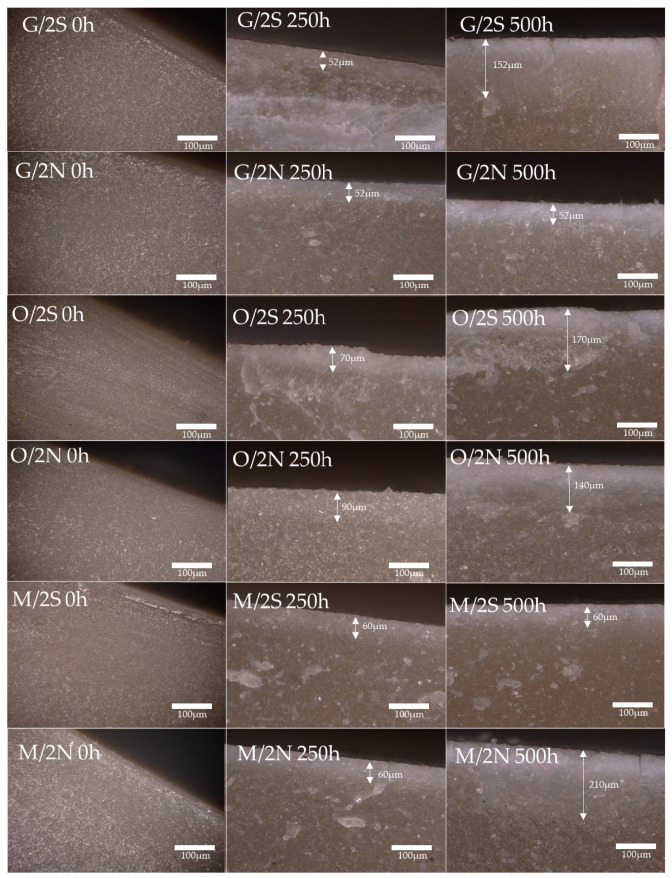
Edge structures of composite breakthroughs before and after placement in UV chamber for 250 and 500 h; silanized systems.

It was also observed how deep the physicochemical changes (degradation-factorsaffected zone) occurred in the composite sample under UV irradiation, as shown in Figure 14 and Figure 15. The reference polylactide already shows changes throughout the volume after 250 h in the aging chamber, which is also evident in the sample conditioned for 500 h, and samples without added silanes (B, B/2S, B/2N) behaved similarly, which can be explained mostly by changes in the level of crystallinity and a decrease in the average molecular weight of PLA [34,35]. On the other hand, in composites filled with silanized diatomaceous earth and with the addition of waxes, the degree of surface degradation increases over time. For composites conditioned for 250 h, the physicochemical changes of the surface occurred no farther than at the 90 μm depth, while, for composites modified with GPTMOS and MTMOS silane, they occuredin the range of 50–60 μm. The system containing OTES silane was characterized by a slightly higher degree of degradation, because, depending on the type of wax used, the depth of changes was about 70 and 90 μm, for O/2S and O/2N, respectively. Only in this case was a slight effect of wax on the degradation rate of the composite observed. After conditioning for 500 h in a UV chamber, the differences became more pronounced. It was observed that the depth of transformation in the composite increased each time, and, for the G/2S and O/2S systems, the progressive depth of degradation and its significantly higher proportion compared to the systems with the addition of natural wax coincides with the results of the microscopic surface analysis discussed above. The silanization of the filler may retard the water diffusion through the filler to the deeper layers of the material, which results in the visible depth of the physicochemical changes.

For the system with the GPTMOS-modified filler and the addition of 2% natural wax, although it has a relatively high level of degradation (62%) after 500 h of aging in a UV chamber (Table 3), this degradation occurs only at a shallow depth, as confirmed by the results of a mechanical analysis of the composites. The results were similar in the case of the O/2N sample.

From the above correlations, it can be concluded that diatomaceous earth causes an increased degradation of polylactide, which is also confirmed by the work of Li T. et al. [36] and Zhang C. et al. [19], while silanization caused a reduction in the negative effect of DE on PLA degradation. The combination of the chemical modification of diatomaceous earth with silanes and the appropriate combination of waxes has the effect of reducing the depth of the degradation of composites.

## 4. Conclusions

From the point of view of the mechanical properties of the filled composites, the modification of polylactide with silanized diatomaceous earth and synthetic wax is more favorable, not only for improving their processing properties. Both fillers and waxes contributed to an increase in the degradation rate of PLA on the surface of the composite, while limiting the depth of influence of the degradation factors, as proven by optical microscopy, which translated into a reduction in the effect of surface cracking and a decrease in mechanical parameters. The diatomite filler, although increasing the water diffusion through the composite and, therefore, accelerating the hydrolytic degradation of PLA, at the same time, works as a UV blocker, decreasing the negative effects of UV irradiation. This complements previous studies, where the positive effect of waxes on the mechanical and processing properties of PLA composites with diatomaceous earth was proven. In addition, it was observed that the physicochemical changes in the surface of composites with natural wax after 500 h in the aging chamber were smaller than with synthetic wax when GPTES- or OTES-modified filler was used. MTMOS caused the opposite effect and the samples with natural wax degraded more strongly. Ecologically, natural waxes leave a smaller carbon footprint because of their production method. The selection of an appropriate wax to improve performance should be dictated by the desire to improve the specific properties of the composites, which change depending on its type. Such a selection of modifiers for polylactide resulted in a significant improvement in the performance of this polymer, due to a reduction in the degree of degradation after conditioning the samples under sunlight. Under natural conditions, degradation was primarily affected by photolytic conditions due to the minimal precipitation during the test, while, under simulated conditions, the moisture level in the system was kept high.

Further research should focus on the further investigation of composites blends containing a different ratio of filler to natural wax for comparative purposes, as well as conducting studies, for example, on the release of components into the environment, while the degradation process occurs in selected composites tested in this study, especially on composites that showed extreme values of degradation depth.

## Figures and Tables

**Table 1:** Injection molding process parameters.

**Temperature [°C]**	**Mold**	**Die**	**Zone 3**	**Zone 2**	**Zone 1**	**Feed**
	80	190	195	200	185	40
**Holding pressure**			**t [s]**	0.0	9.0	
			**P [bar]**	500	1500	
**Clamping force [kN]**			**Holding time [s]**	**Coolig time [s]**	**Screw diameter [mm]**	
800			9.0	50.0	25.0	

**Table 2:** The prepared, injection-molded composite systems.

Full Name	Short Name
PLA	PLA
PLA/DE	B
PLA/DE + 1% wt. SW	B/1S
PLA/DE + 2% wt. SW	B/2S
PLA/DE + 1% wt. NW	B/1N
PLA/DE + 2% wt. NW	B/2N
PLA/DE + 1% wt. GPTMOS + 1% wt. SW	G/1S
PLA/DE + 1% wt. GPTMOS + 2% wt. SW	G/2S
PLA/DE + 1% wt. GPTMOS + 1% wt. NW	G/1N
PLA/DE + 1% wt. GPTMOS + 2% wt. NW	G/2N
PLA/DE + 1% wt. OTES + 1% wt. SW	O/1S
PLA/DE + 1% wt. OTES + 2% wt. SW	O/2S
PLA/DE + 1% wt. OTES + 1% wt. NW	O/1N
PLA/DE + 1% wt. OTES + 2% wt. NW	O/2N
PLA/DE + 1% wt. MTMOS + 1% wt. SW	M/1S
PLA/DE + 1% wt. MTMOS + 2% wt. SW	M/2S
PLA/DE + 1% wt. MTMOS + 1% wt. NW	M/1N
PLA/DE + 1% wt. MTMOS + 2% wt. NW	M/2N

DE—diatomaceous earth; PLA/DE—PLA + 25% wt. DE; SW—synthetic wax; NW—natural wax; GPTMOS—3-glicydoxypropyltrimethoxysilane; OTES—n-oktyltriehtoxysilane; MTMOS—metylotrimethoxysilane.

**Table 3:** Summary of the molecular weights of the polymers tested and the calculated percentage of degradation.

Sample Name	Mn (250 h)	Mn (500 h)	Mn (NC)	Mw (250 h)	Mw (500 h)	Mw (NC)	%Degradation 250 h ^[a]^	%Degradation 500 h ^[a]^	%Degradation NC ^[a]^
PLA	82,600	55,100	26,000	148,500	126,000	84,100	20.7	34.9	58.5
B	27,000	37,600	28,800	63,700	96,000	103,400	50.4	65.9	48.9
B/1S	71,400	46,900	33,600	116,400	105,900	108,400	37.8	45.3	46.5
B/2S	40,900	34,500	56,900	93,700	81,200	143,000	49.9	58.1	29.4
B/1N	45,100	36,400	38,300	94,800	82,800	9100	49.4	57.2	95.5
B/2N	43,100	36,300	33,300	99,600	82,800	116,300	46.8	57.2	42.6
G/1S	47,500	20,600	44,300	99,300	52,500	133,200	47.0	72.9	34.2
G/2S	35,100	16,400	45,700	90,500	39,200	119,000	51.6	79.8	41.3
G/1N	43,500	20,900	39,700	82,600	64,800	90,100	55.9	66.5	55.5
G/2N	31,500	31,600	18,400	70,300	74,000	56,600	61.8	62.5	72.1
O/1S	28,600	22,200	27,700	65,400	54,700	110,300	65.1	71.8	45.5
O/2S	33,000	25,100	17,300	74,600	54,900	103,800	60.1	71.6	48.7
O/1N	47,200	30,600	65,700	109,400	75,500	121,000	41.5	61.0	40.3
O/2N	61,100	27,300	75,500	90,800	74,900	146,100	51.5	61.3	27.9
M/1S	29,100	19,400	67,400	73,300	53,600	111,400	60.8	72.3	45.0
M/2S	24,500	17,600	42,000	58,800	52,800	143,800	68.6	72.7	29.0
M/1N	25,600	22,500	39,200	60,900	56,300	118,700	67.5	70.9	41.4
M/2N	44,300	24,500	43,100	101,300	72,600	120,400	45.9	62.5	40.2

NC—natural conditions; 250 h, 500 h—UV chamber; Mn—number average molecular weight; Mw—average molecular weight. ^[a]^—degradation % calculated from Mw change.

## Data Availability

Data are contained within the article.
